# Recent findings from the anaphylaxis registry: Where are we, where do we want to go? 

**DOI:** 10.5414/ALX02529E

**Published:** 2025-03-06

**Authors:** Margitta Worm, Sabine Dölle-Bierke, Lea Faust, Veronika Höfer

**Affiliations:** Allergology and Immunology, Clinic for Dermatology, Venereology and Allergology, Campus Charité Mitte, Universitätsmedizin Berlin, Berlin, Germany

**Keywords:** anaphylaxis, elicitors, anaphylaxis registry, time trends, rare allergens

## Abstract

The anaphylaxis registry collects structured data across Europe regarding elicitors, accompanying circumstances and other diseases as well as the treatment of severe allergic reactions. By March 2024, 16,988 cases had been registered, of which ~ 1/3 were children and 2/3 were adults. Among children, boys are most frequent affected, while among adults, women are most frequently affected. The most common elicitors depend on age and continue to be dominated by the food allergens in children and by insect venom in adults. The occurrence of anaphylaxis without skin symptoms is not uncommon and occurs in children in the range of 5%, regardless of the trigger, while in adults the frequency is generally higher (up to 15%) and there are trigger-dependent differences. The analysis of the rare food allergens that trigger anaphylaxis shows, for example, spices such as saffron and sumac and vegetables such as chicory or spring onions. One case was elicited by a mealworm. Food shows an increase in triggering anaphylaxis in children. The trigger spectrum of foods is large and relevant due to the increasing plant-based diets. Rare allergens can cause anaphylaxis and should be further considered and monitored in the coming years to determine their frequency as triggers of severe reactions.

## Introduction 

The anaphylaxis registry was initiated in 2007, initially in German-speaking countries (Germany, Austria, and Switzerland) and from 2011 across Europe. The aim of the anaphylaxis registry is to record severe allergic reactions in terms of triggers, accompanying circumstances and diseases as well as treatment in a standardized manner. The register records patients who have an involvement of two organ systems, e.g., respiratory and/or cardiovascular manifestations in addition to skin symptoms. Allergy-specialized centers that care for both children and/or adults take part. 

The first analyses of the register data have shown that the most common triggers (insect venom, medication, and food) are age-dependent and that grade II and III reactions were predominantly reported [1]. In further analyses, we were able to identify certain cofactors for anaphylaxis (e.g., age, mastocytosis, and certain medications) that can increase the risk of a severe reaction [2]. Elicitor-specific analyses considering the three main elicitor groups (insects, food, and drugs), showed that the clinical symptoms can vary depending on age and elicitor [3, 4, 5]. 

In this article, we would like to report the current status of the cases reported to the anaphylaxis registry up to 2024 and present the temporal developments of the relative frequencies for the most common triggers. An important finding from the register data is the observation that severe anaphylaxis can often manifest itself in adults, but also in children, even without skin symptoms. Moreover, the spectrum of rare food allergens is analyzed in more detail. 

## Materials and methods 

The present analysis is based on data from the anaphylaxis registry reported up to March 2024. Since 2007, the anaphylaxis registry has been collecting data on age, gender, triggers, and symptoms of anaphylaxis using a standardized online questionnaire [1]. The data was entered by trained staff from the participating allergy centers after the patients had undergone an allergy assessment. Annual quality controls are carried out, and only cases that meet the criteria for anaphylaxis based on the definition of the National Institutes of Allergy and Infectious Diseases/Food Allergy and Anaphylaxis Network (NIAID/FAAN) [6] are taken into account for the data analyses. 

## Results 

### Trigger frequencies in children and adults 

**Figure 1:**
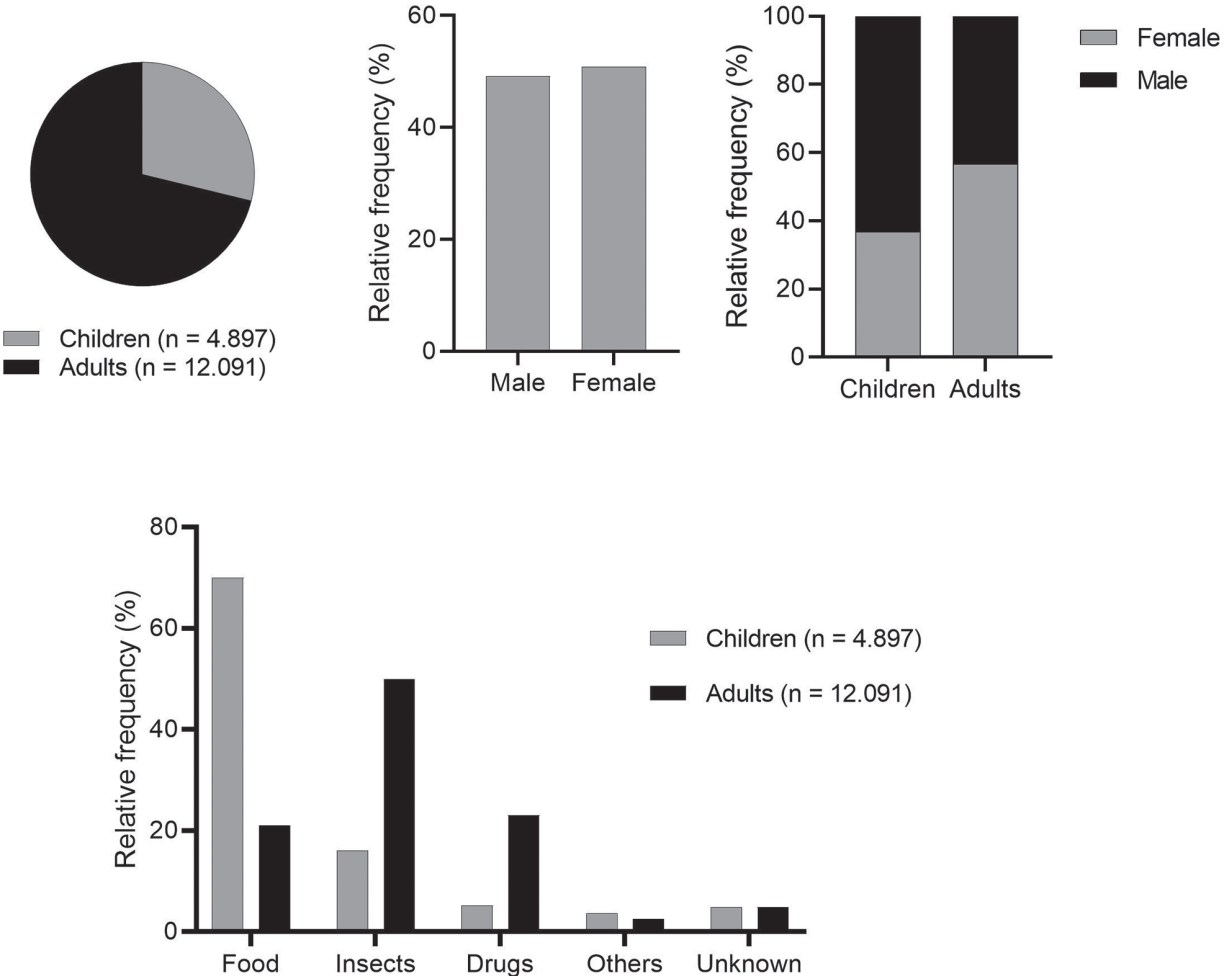
Age, gender and trigger distribution of the cases reported to the anaphylaxis registry from 2014 until 2024.

By March 2024, 16,988 cases had been registered according to the inclusion criteria for the anaphylaxis registry. Of these, ~ 30% were children (n = 4,897) and ~ 70% were adults (n = 12,091), with the gender distribution in the overall cohort being balanced (Figure 1). However, in the children’s group, there was a predominance of the male sex (63.1%), and in the adults there were more female patients (56.6%). Regarding the most common trigger groups, previous results are confirmed and show that food is the most common trigger in children (70%), followed by insects (16%) and medications (5.2%). In adults, insect venom is still the most common trigger with a relative frequency of 50%, followed by medications (23%) and food (21%). 

### Relative frequency of elicitors from 2014 to 2024 for food, medications, and insects 

**Figure 2:**
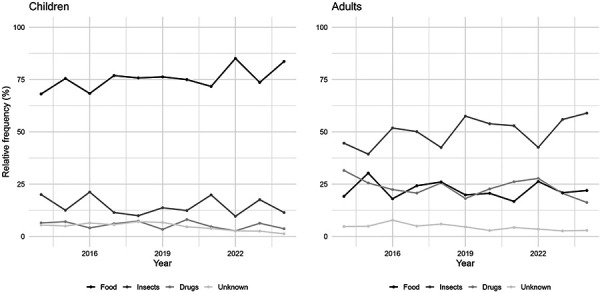
Time course of the relative frequencies of the most common anaphylaxis trigger groups.

The analysis of the frequencies for the trigger groups from 2014 to 2024 shows an increase in food for the children’s group, while insects and medications as triggers of anaphylaxis have been stable in children over the last 10 years (Figure 2). In adults, insect venoms are still the leading trigger group and have shown an increasing frequency in the last 2 years, although there are probably fluctuations every year due to climate change and other factors. In contrast, in adults, there is a decreasing trend in medication as a trigger, while food as a trigger has remained stable. Interestingly, for children and adults, there is a clear decreasing average frequency trend in the category of “unknown trigger”. 

### Frequencies of cases without skin symptoms in children and adults 

**Figure 3:**
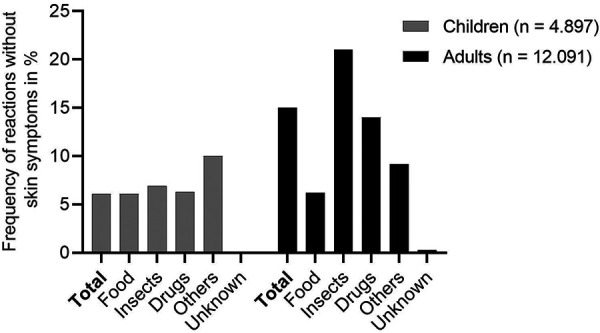
Relative frequency of anaphylaxis without skin involvement differentiated by age and elicitor groups.

In previous analyses, we have shown that there is a patient subgroup that does not develop skin symptoms despite anaphylaxis [5]. We therefore looked at this again in the current overall cohort with large numbers of patients (Figure 3). The data show that in the total group of 12,091 from the adult group, 1,759 (15%) patients did not experience any skin symptoms in the context of the anaphylactic reaction. The largest group without skin symptoms suffer from insect venom (21%) and drug (14%) induced anaphylaxis but less frequently in the context of food-triggered reactions. Such a group of patients without skin symptoms can also be identified for children and adolescents, but the absolute number is significantly lower (299 patients) and reaches only 6.1%. For this cohort, the elicitor-specific analysis did not reveal any relevant differences. 

### Spices and other food allergens as rare triggers 

**Figure 4:**
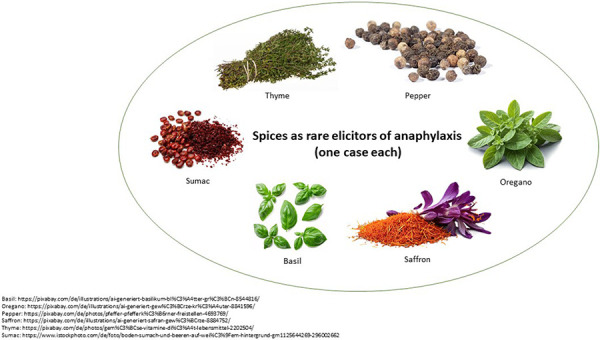
Spices as a rare trigger of anaphylaxis with one reported case each.

Although the consideration of the most common triggers of anaphylaxis is relevant, it is just as important to examine rare triggers in order to identify, e.g., potentially new allergens. The analysis of rare triggers of food-induced anaphylaxis revealed a broad spectrum and included mainly fruit and vegetables but also spices and, in individual cases, substances such as agar-agar, baobab powder, tiger nuts, or psyllium husks. It is interesting to note that spices such as basil, oregano, pepper, thyme, saffron, and sumac have been identified to trigger reactions in individual cases (Figure 4). Among the triggers in the context of the vegetable subgroup, anaphylaxis was reported in individual cases (not shown) to be related to parsnips, chicory, spring onions, radishes, broccoli, and kohlrabi. One case was reported to be induced by a mealworm (not shown). Overall, the data show that a careful trigger assessment and clarification should also be carried out with regard to rare allergens in food anaphylaxis patients. 

## Discussion 

In this manuscript, we present the latest data from the anaphylaxis registry, which now includes a cohort of 16,988 patient cases. Previous data on the most common triggers could be confirmed [1], although compared to earlier analyses, the relative proportion of food as a trigger has increased in children but not in adults. This goes hand in hand with current data from the literature, which show that, for example, hospitalizations as a result of a food allergy in children have increased in the last 10 years [7]. 

It is interesting to note that the category of “unknown triggers” has decreased in both children and adults in the last 10 years. The rate for children now reaches 3.1% and for adults 2.8% (compared to 2014: 5.2 and 4.5%, respectively). This is most likely due to improved diagnostics in the area of food allergy. Molecular allergy diagnostics in particular have made it possible to improve the sensitivity for detecting IgE sensitization, at least for certain allergens. Furthermore, new allergens are constantly being identified that may be the triggering allergen for some patients. One example is Ara h 18, which has recently become commercially available [8]. Ara h 18 belongs to the cyclophilin family. In this protein family the structure is highly conserved and they are present in a wide variety of plant foods, such as tomatoes and various others [9]. 14% of peanut-sensitized individuals with positive sIgE to peanut extract and negative sIgE values to all available recombinant allergen components of peanut and cross-reactive carbohydrate determinants (CCD) [10] showed Ara h 18 sensitization. 

Of high relevance is our observation of a relevant frequency (up to 15% in adults) of anaphylactic reactions without skin symptoms. This frequency is about 3 times higher in adults than in children and also seems to depend on the trigger. The highest frequency was identified among patients with anaphylaxis to insect venom and was significantly less frequent in food-induced anaphylaxis (6%), but still common. 

This phenomenon is particularly known for insect venom allergy, although the mechanisms behind this are unclear. It must be taken into account that it is possible that skin symptoms may only appear for a very short time, may be concealed by clothing or may even appear out of the diagnostic frame. In addition, further research into the pathophysiology of mast cells in these patient groups would be of scientific interest. The lack of skin involvement is not only observed in adults, but also in children, although less frequently. However, we did not determine a trigger dependency among children. 

The analysis of rare triggers of anaphylaxis make it possible to identify, e.g., new foods with allergenic potential. The spectrum of triggers of rare allergens is broad and includes all known subgroups. In particular with regard to spices, we also find severe anaphylactic reactions in individual cases. The diversified diet in today’s society with complex eating habits has led to the conception of an increasing variety of spices. In the future, it will be of great interest to prospectively follow these findings. 

So far, the triggering allergens have been described for individual subgroups of spices such as basil and oregano, but also for more exotic species such as saffron [11]. Profilins and lipid transfer proteins have been identified as triggering allergens [12, 13]. Sumac (*Rhus coriaria*) is a popular spice in Turkish and Arabic cuisine and is botanically closely related to cashew [14]. The tiger nut, a sedge plant from the Mediterranean region, is also currently described in the literature as a rare trigger of anaphylaxis [15]. While the consumption of edible insects is still rather uncommon in Europe, recent studies indicate that the consumption of mealworms (*Tenebrio molitor*) can induce anaphylactic reactions in patients with crustacean allergy [16]. Given the increasing relevance of insects as a source of protein, we suspect that these will have to be considered as potential allergens in food in the future, although we so far observed only one case in the register that was triggered by edible insects. 

## Conclusion 

Food is increasingly considered to be the trigger of anaphylaxis in children, while in adults it is insect venoms. Due to the diversity of diets and increasingly plant-based diets, the spectrum of triggers in the area of food has expanded. Skin symptoms as a central symptom of anaphylaxis may be absent in children and adults, making differential diagnostic procedures more difficult. Rare allergens such as spices or edible insects can cause anaphylaxis in individual cases and should be further monitored in the coming years with regard to their frequency as triggers of severe anaphylactic reactions. 

## Acknowledgment 

The authors would like to thank the study staff of all actively participating registry centers: J. Grünhagen, P. Globig, F. Ruëff, T. Biedermann, K. Brockow (Munich, Germany), K. Hartmann (Basel, Switzerland), K. Scherer (Aarau, Switzerland), C. Pföhler (Homburg, Germany), R. Treudler (now Berlin, formerly Leipzig, Germany), F. Prenzel (Leipzig, Germany), N. Wagner (Erlangen, Germany), A. Köhli (Baden, Switzerland), T. Hawranek and R. Lang and W. Eder (Salzburg, Austria), A. Bauer (Dresden, Germany), M. Bilo (Ancona, Italy), D. Sabouraud-Leclerc, JM. Renaudin, S. Tscheiller and all members of the Allergy Vigilance Network (France), N. Papadopoulus (Athens, Greece), L. Lange (Bonn, Germany), A. Kleinheinz (Buxtehude, Germany), A. Möser (Jena, Germany), B. Wedi (Hannover, Germany),H. Dickel (Bochum, Germany), E. Rietschel (Cologne, Germany), N. Reider (Innsbruck, Austria), B. Kreft (Halle, Germany), K. Nemat (Dresden, Germany), T. Kinaciyan and Z. Szepfalusi (Vienna, Austria), U. Rabe (Treuenbritzen, Germany), P. Schmid-Grendelmeier, M. Hoernes, J. Trück (Zurich, Switzerland), W. Brosi (Würzburg, Germany), R. Bruns (Greifswald, Germany), M. Polz (Rüsselsheim, Germany), S. Thies (Schwedt, Germany), A. Nordwig (Dresden, Germany), S. Lay, (Wangen im Allgäu, Germany), A. Henschel, K. Parasher, K. Beyer, U. Staden (Berlin, Germany), K. Schäkel (Heidelberg, Germany), M. Kurowski (Lodz, Poland), M. Fernandez-Rivas, T. de Vincente (Madrid, Spain), T. Mustakov (Sofia, Bulgaria), C. Kemen (Hamburg, Germany), J. Seidenberg and H. Köster (Oldenburg, Germany), V. Cardona, O. Dominguez, R. Munoz Cano, R.L. Bellfill (Barcelona, Spain), S. Hämmerling (Heidelberg, Germany), B. Garcia (Pamplona, Spain), I. Poziomkowska-Gęsicka (Stettin, Poland), S. Büsing (Osnabrück, Germany), G. Christoff (Sofia, Bulgaria), U. Jappe (Borstel, Germany), S. Müller (Freiburg, Germany), H. Straube (Darmstadt, Germany), C. Vogelberg (Dresden, Germany), J. Hourihane (Dublin, Ireland), A. Muraro (Padua, Italy), S. Altrichter (Linz, Austria), A. Fiocchi (Rome, Italy), C. Feder (Jena, Germany), K. Beyer (Berlin, Germany), F. Horak (Vienna, Austria), H. Ott (Hannover, Germany), E. Cichocka-Jarosz (Krakow, Poland), L. Ensina (Sao Paulo, Brazil), J. Meister (Aue, Germany), P. Stock, S. Hompes (Hamburg, Germany), E. Hamelmann (Bielefeld, Germany), T. Spindler (Scheideegg, Germany), I. Neustädter (Nuremberg, Germany), I. Maris (Cork, Ireland), S. Hofmann (Wuppertal, Germany), P. Turner (London, England), G. Heine (Kiel, Germany), D. Deleanu (Cluj-Napoca, Romania), T. Jakob (Giessen, Germany), T. Yavuz (Bonn, Germany), R. Munoz Cano and R. Lleonart Bellfill (Barcelona, Spain), J.N.G. Oude Elberink (Groningen, Netherlands), V. Vukicevic (Zagreb, Croatia). 

## Authors’ contributions 

Margitta Worm: Conceptualized and supervised the study, wrote the manuscript. Sabine Dölle-Bierke: Assisted in writing the manuscript, reviewed the manuscript. Lea Faust: Assisted in writing the manuscript, reviewed the manuscript. Veronika Höfer: performed the statistical analysis, reviewed the manuscript. 

## Funding 

The Anaphylaxis registry is supported by the Network for Online Registration of Anaphylaxis NORA e.V. 

## Conflict of interest 

M. Worm declares the receipt of honoraria or consultation fees by the following companies: Abbvie, Aimmune, AKM, ALK, Allergopharma, Almirall, Amgen, AstraZeneca, Bayer, Bencard, Bioprojet Pharma, BMS, Boehringer Ingelheim, Cerner Enviza, Dustri, Galderma, GSK, Infectopharm, Leo Pharma, Lilly, med update, Meda/Mylan/Viatris, Novartis, Octapharma, Pfizer, Sanofi and streamedup.

S. Dölle-Bierke, L. Faust und V. Höfer declare no conflict of interest.
